# A rapid method to screen putative mRNA targets of any known microRNA

**DOI:** 10.1186/1743-422X-8-8

**Published:** 2011-01-11

**Authors:** Yujing Huang, Ying Qi, Qiang Ruan, Yanping Ma, Rong He, Yaohua Ji, Zhengrong Sun

**Affiliations:** 1 Virus Laboratory, the Affiliated Shengjing Hospital, China Medical University, 110004 Shenyang, Liaoning, PR China

## Abstract

**Background:**

microRNAs (miRNAs) are a group of regulatory RNAs that regulate gene expression by binding to specific sequences on target mRNAs. However, functional identification of mRNA targets is usually difficult and time consuming. Here we report hybrid-PCR as a new and rapid approach to screen putative mRNA targets in vitro.

**Results:**

Fifteen putative target mRNAs for human cytomegalovirus (HCMV) miR-UL112-1, including previously confirmed HCMV IE72, were identified from mRNA-derived cDNAs using hybrid-PCR. Moreover, we randomly validated six different target candidates by luciferase reporter assays, and confirmed that their luciferase activities were down-regulated with co-transfection of HCMV miR-UL112-1.

**Conclusions:**

Our study demonstrated that hybrid-PCR is an effective and rapid approach for screening putative miRNA targets, with much more advantage of simplicity, low cost, and ease of implementation.

## Background

MicroRNAs (miRNAs) are the most studied non-coding RNAs in recent years. miRNAs are 17- to 30-nucleotide RNAs that are ubiquitously expressed in plants and animals. They regulate gene expression at the posttranscriptional level [1,2] and act as key regulators in diverse regulatory pathways, including early development, cell differentiation, cell proliferation, metabolism and apoptosis [3-6]. miRNAs binding to target mRNAs often leads to blockade of translation or degradation of the target mRNAs. Identification of target mRNAs is essential for understanding the biological functions of miRNAs. miRNAs from plants induce direct cleavage and degradation by binding to the target sequences with perfect base pairing. Targets of mammalian miRNAs are often difficult to predict, because few of them match to their target mRNAs perfectly [7]. Their miRNA:mRNA duplexes often contain several mismatches, gaps and G:U base pairs in many positions [8]. While it is known that a so-called miRNA "seed region" (nucleotide 2-7 at the 5'-end of miRNA) is the most important determinant for target specificity [9]. miRNA-mediated repression often depends on perfect or near-perfect base pairing of seed regions to their targets [10,11].

A conventional way to search for miRNA targets is by using bioinformatics. The classical model for specific miRNA target recognition by most algorithms was mainly depended on (a) the detection of seed matches and (b) thermodynamic stability of miRNA:mRNA duplexes. Different algorithms always produce divergent results [1,12-14]. In addition, much work has been done to develop biochemical tools to identify miRNA targets, such as HITS-CHIP [15-17] and microarray technique. Those biochemical tools have been proven to be useful in miRNA targets research, but they are not widely applied because their processes are too complicated. In this study, we reported a rapid experimental approach for screening putative target mRNAs of any known miRNA.

Polymerase Chain Reaction (PCR) is widely held as one of the most important experimental methods in molecular biology. In addition to being complementary, the stability of primer-template hybridization is essential for successful PCR reactions. These requirements are also true for miRNA target recognition. Thus we thought a pool of information of target mRNAs might be established in the manner of individually designed PCR to screen putative targets of miRNAs. Because the new screening approach worked mainly in the form of PCR, we named it hybrid-PCR in our study.

To investigate whether hybrid-PCR could functionally identify putative miRNA targets, human cytomegalovirus (HCMV) miR-UL112-1 was selected as the research object in our study. It was difficult to recognize target mRNAs from HCMV genome by bioinformatics, because too little information of HCMV mRNA sequences could be obtained from any database. Some functional target mRNAs of miR-UL112-1 had been identified recently, thus the efficiency of hybrid-PCR in screening putative targets could be confirmed by using those targets as references.

## Results

**Figure 1:**
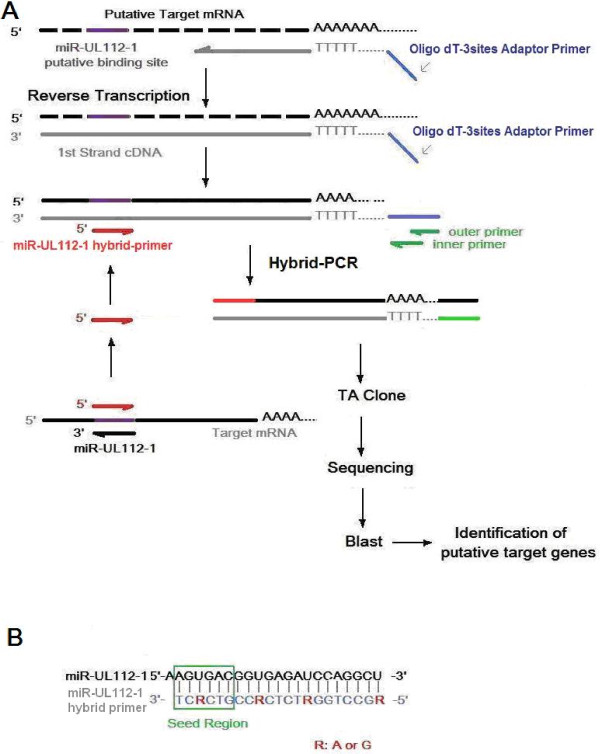
**Protocol of hybrid-PCR**. (A) Schematic presentation of principle and process designed for hybrid-PCR. (B) Diagram showing sequences of miR-UL112-1 and miR-UL112-1 hybrid primer. Positions marked by Red R meant random insertions of A or G. Seed region was indicated by green box surrounding nucleotide 2-7 of miR-UL112-1.

**Figure 2:**
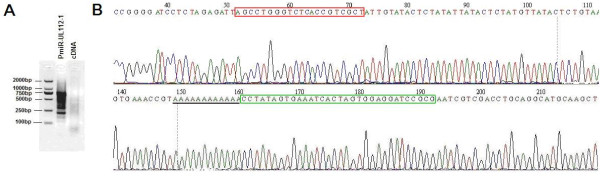
**Results of hybrid-PCR**. (A) Hybrid-PCR was carried out as described. Product of hybrid-PCR (PmiR-UL112-1) and mRNA-derived cDNA (cDNA) were electrophoresis on 3% agarose gel with DL2000 alongside. (B) Partial chromatogram of clone B29, which was identified containing HCMV IE72 specific sequence. Sequence of miR-UL112-1 hybrid-primer was indicated in red box, and inner primer binding site was indicated in green box. PolyA sequence was down lined in black.

miRNAs play the role of posttranscriptional regulation by binding to target mRNAs, hence the target sequences were screened among mRNA-derived cDNAs in hybrid-PCR. An oligo dT-3 sites adaptor primer was introduced into 5'-terminal of mRNA-derived cDNA during reverse transcription (Figure 1A). This primer distinguished the mRNA-derived cDNAs effectively from other DNAs or RNAs in amplification. miRNA specific hybrid-primer was designed according to the miRNA sequence. The reverse and complementary sequence of the seed region of miRNA was lacated at the 3'terminal of the hybrid-primer. Hybrid-PCR was projected as semi-nested PCR using the hybrid-primer and the outer/inner primers homologous to the oligo dT-3 sites adaptor primer. Specificity of target mRNA of a given miRNA was determined by hybridization of the hybrid-primer to the sequence of mRNA-derived cDNA. A low annealing temperature of 37°C was applied in the first round amplification, so as to make hybrid-primer hybridize with putative target sequences in a condition similar to core body temperature. Then a second round PCR with higher annealing temperature of 55°C was followed for further specific amplification of sequences from putative target mRNAs. Extension was long enough to avoid incomplete amplification. The products of amplification were variable in length (Figure 2A).

To acquire the actual sequences from miR-UL112-1 putative target mRNAs, products of hybrid-PCR were purified, cloned into T-vector and sequenced. Fifty-four sequences were obtained successfully in our study. Hybrid-primer sequences and polyA structure were confirmed for a complete extremity of mRNA. mRNA specific sequences located between hybrid-primer and polyA were intercepted and used to blast online to identify their host genes. Fifty-one sequences matched sequences in GenBank and their host mRNAs were identified successfully. The other three were not identified because their specific sequences (4-6 nucleotides) were too short. Overall 15 putative target mRNAs of HCMV miR-UL112-1 were obtained. Detailed information is reported in Table 1. HCMV immediate early protein (IE72) gene, a confirmed miR-UL112-1 target gene [18], was identified in our result (Table 1 and Figure 2B). The miR-UL112-1 binding sites of three identified putative target mRNAs were not located in 3'UTR (Table 1). An extensive set of binding sites was identified in our result, such as coding sequence. Perfect base pairing within seed region was not observed in all sequences.

**Table 1:** Putative target mRNAs of HCMV miR-UL112-1 identified by hybrid-PCR

Putative target mRNAs		Number of clones	In 3'UTR	Complementary to Seed Region	Predicted by TargetScan	Repeoted before
**mRNA encoded**	**Accession No**.					

HCMV immediate early protein (IE72)^a^	M26973.1	1	+	+		+
HCMV UL17/18 ^a^	AC146906.1	1		+		
Homo sapiens heat shock protein, alpha-crystallin-related,B6	NM_144617.1	8	+			
Homo sapiens CCAAT/enhancer binding protein (C/EBP)	NM_005195.3	5	+	+	+	
Homo sapiens NADH dehydrogenase subunit 5 (MTND5)	AF339085.1	2	+	+		
Homo sapiens microfibrillar-associated protein 1 (MFAP1)	NM_005926.2	2		+		
Homo sapiens mRNA for putative NFkB activating protein ^a^	AB097011.1	1	+	+		
Homo sapiens interleukin 32 ^a^	NM_001012631.1	1	+			
Homo sapiens ribosomal protein S18	NM_022551.2	6				
Homo sapiens ribosomal protein L7a ^a^	BC032533.1	12	+	+		
Homo sapiens spermine oxidase	NM_175842.1	3	+			
Homo sapiens transportin 1 ^a^	NM_002270.3	3	+	+	+	
Homo sapiens HSPC193	NM_001145104.1	1	+			
Homo sapiens z-cop	AF086911.1	1	+	+		
Homo sapiens zinc finger protein 36 ^a^	NM_004926.2	4	+	+		

Note: Genes conformed to the descriptions were marked by "+"in columns. Genes marked by "a" were validated by luciferase reporter assays.

**Figure 3:**
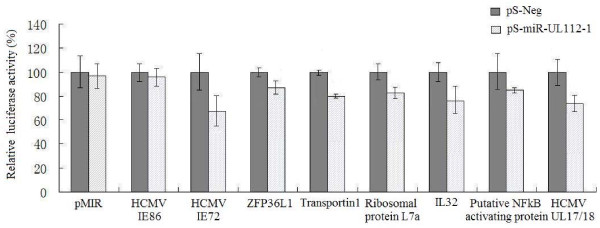
**HCMV miR-UL112-1-mediated repression of luciferase reporter gene activity**. Putative target sequences were validated for their ability to inhibit expression of a luciferase reporter construct in the presence of HCMV miR-UL112-1 (pS-UL112-1) respectively. Results were shown as percentage expression of negative control sample (pS-Neg) following correction for transfection levels according to control renilla luciferase expression. Values are means ± standard deviations for triplicate samples.

To determine whether the putative binding sequences obtained by hybrid-PCR represent functional target sites for miR-UL112-1, we validated a number of mRNAs using another experimental approach. Six putative binding mRNAs were randomly chosen from our results above, including those whose target sites were not located in 3'UTR (HCMV UL17/18) or complementary perfectly to seed region (Homo sapiens interleukin 32). The target binding sequences along with flanking sequences were cloned downstream into a luciferase reporter construct pMIR respectively. So was the 3'UTR of HCMV IE72 mRNA, which was used as a positive control in luciferase reporter assays. The 3'UTR of HCMV IE86 mRNA does not contain the miR-UL112-1 target sequence [18]. A pMIR construct containing the 3'UTR of IE86 provided an ideal negative control in luciferase reporter assays. Compared to the pSilencer negative control group, co-transfection of HCMV miR-UL112-1 with pMIR containing candidate target sequences all led to a decrease in luciferase activity (Figure 3). However, expression of miR-UL112-1 caused only a minor reduction in luciferase activity of pMIR containing the 3'UTR of IE86. These data demonstrate that the putative binding sites that have been validated in our study could indeed be recognized by HCMV miR-UL112-1.

**Figure 4:**
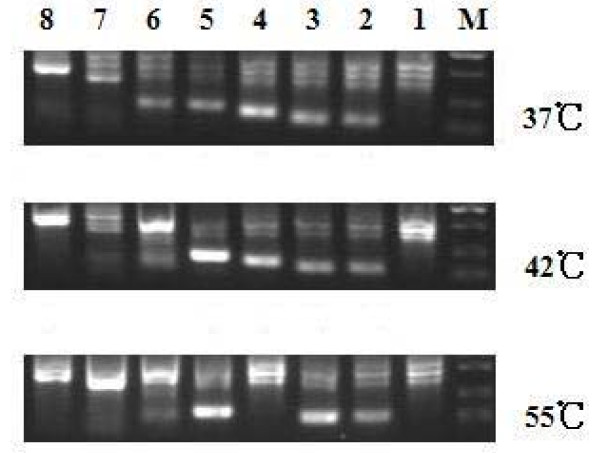
**Identification of seven validated target genes among hybrid-PCR products with different initial annealing temperature**. Seven validated target sequences (including IE72) were identified among those hybrid-PCR products by an additional amplification with specific primers of target sequence. M, DL2000; lane 1, negative control; lane 2, mRNA of HCMV IE72; lane 3, mRNA of zinc finger protein 36; lane 4, mRNA of transportin 1; lane 5, mRNA of ribosomal protein L7a; lane 6, mRNA of interleukin 32; lane 7, mRNA for putative NFkB activating protein; lane 8, mRNA of HCMV UL17/18.

Hybrid-PCR was designed to identify target sequences of a miRNA by nearly perfect base pairing of seed region through a low annealing temperature in the initial PCR. 37°C was used as the initial annealing temperature because it was close to the core body temperature, which was considered similar to the physiological hybridization environment. To determine whether different initial annealing temperature could affect the results of hybrid-PCR, a series of amplifications with different initial annealing temperatures (37°C, 42°C and 55°C) was processed. Then, gene specific primers were used to identify the seven validated target sequences (including IE72) among those products. As shown in Figure 4, the number of target sequences identified was decreased along with the increase of initial annealing temperature, while there was no correlativity observed between the target sequences identified by PCR with different initial annealing temperatures and the down regulation abilities of luciferase activities.

## Discussion

It's known that perfect complement was not essential for functional binding of a miRNA to a target sequence. However, binding within bases 2 to 7 of the miRNA known as seed region is considered particularly important. Hybrid-PCR was carried out using a miRNA-specific primer containing the reverse and complementary sequence of the seed region of a given miRNA at the 3' terminal Putative target sequences could be acquired by hybrid-PCR relying on imperfect base pairing through a low annealing temperature (37°C) in the initial PCR. This initial annealing temperature was approved to be crucial by a series of amplifications with different initial annealing temperatures. As a method for screening of putative target mRNAs of a given miRNA, quantity of information identified by the Hybrid-PCR should be a key point. Our results indicated that some information important would be missed if the annealing temperature was higher than 37°C in the initial PCR step.

Prediction of miRNA targets by Bioinformatics method depends on a genome-wide database of all cellular mRNAs, but such a database, especially that of viruses, is still not available. Three prediction algorithms (targetScan, Miranda and pictar) are most widely used in miRNA target research. However, only targetScan (http://genes.mit.edu/targetscan) could be used in our research. There was no information of HCMV mRNA recruited in the prediction algorithms Miranda and pictar, of which the prediction of target mRNAs was depended on the accomplishment of mRNA database. The lack of bioinformatics limits target prediction of miRNAs in species such as viruses. Hybrid-PCR could catch the targets of a known miRNA directly from mRNA-derived cDNAs. This method is useful for the identification of miRNA binding sites within poorly annotated mRNAs such as those expressed by HCMV.

The expression of miRNAs often shows temporality and tissue specificity, but the prediction of targets by bioinformatics method can not be modulated according to those characteristics. Expressions of genes are various in different cells, even in different stage of the same cell. Only mRNAs in the miRNA expressing cells could be the candidate targets of the miRNA. Based on genome-wide database of all cellular mRNAs, huge unexpressed mRNAs in certain cells will be predicted to be targets by Bioinformatics. Hybrid-PCR has much more flexibility and can be used to identify target mRNAs for a miRNA from any kind of cells at different development stages or from different tissues. Hybrid-PCR can identify the mRNAs only expressed in certain cells or cell stages. Two of the fifteen mRNAs identified in our study are predicted by targetScan (Table 1). Therefore, more miRNA targets might be identified by hybrid-PCR rather than by miRNA target prediction algorithms.

## Conclusions

In summary, hybrid-PCR is a simple and effective method to screen putative target mRNAs of a known miRNA. Clear advantages of this method are its simplicity, low cost, and ease of implementation. Target mRNA candidates can be obtained through hybrid-PCR from any kind of cells at different development stages or from different tissues. Hybrid-PCR can be used as a quick screen tool in miRNA research, although more experimental validations are needed in further study.

## Methods

### Virus preparation and Cell culture

Clinical strain of HCMV named Han was isolated from a urine sample of a 5-month-old infant hospitalized in Shengjing Hospital of China Medical University. Han strain was passaged six times in human embryonic lung fibroblasts (HELF) maintained in 1640 medium supplemented with 2% fetal bovine serum (FBS), 100 units/ml penicillin and 100 units/ml streptomycin. HELF cells were inoculated with Han strain at a multiplicity of infection (m.o.i.) of 3-5 PFU per cell. Infection was carried out under immediate early condition (1 h preinfection then 24 h in 200 μg/ml cycloheximide), and cells were harvested for further RNA isolation.

Human embryonic kidney cells (HEK 293) were maintained in Dulbecco's modified Eagle medium (DMEM) containing 10% FBS, 100 units/ml penicillin, 100 units/ml streptomycin and 2 mM L-glutamine (Invitrogen).

### RNA isolation and mRNA purification

Total RNA was isolated from approximately 10^7 ^HCMV infected HELF cells using Trizol agent (QIAGEN), and then processed using Oligotex mRNA Kits (QIAGEN) according to the protocol. mRNA was dissolved in 200 μl RNase free H_2_O and treated by TURBO DNA-free™ Kit (Ambion). The integrity of the mRNA was analyzed on 1% agarose gel electrophoresis alongside RNA marker.

### Primer design for hybrid-PCR

A miR-UL112-1-specific primer was designed for hybrid-PCR. A reversal and complementary sequence of HCMV miR-UL112-1 gene was generated for miR-UL112-1 hybrid-primer, which was inferred to recognize the putative binding sites of miR-UL112-1 located in mRNAs (Figure 1B). The seed region of HCMV miR-UL112-1 was correspondingly located in the 3'-terminal of hybrid-primer. The last base T was considered not essential for perfect complement and deleted from the 3'-terminal of hybrid-primer. Since G:U pairs are allowed for the miRNA:mRNA duplexes, the miR-UL112-1 hybrid-primer was synthesized as a compatible primer: Adenines (A) located in miR-UL112-1 hybrid-primer were substituted by random insertions of adenines (A) or guanines (G).

### Hybrid-PCR and sequencing

Reverse transcription was performed with 1 μg mRNA using 3'-Full RACE Core Set (TaKaRa). The first-strand cDNA was synthesized as a template for further PCR amplification, with an oligo dT-3 site adaptor primer introduced into its 5'-terminal. Hybrid-PCR was then carried out using nested primers which were homologous to the Oligo dT-3 sites adaptor primer (outer primer: 5'-TACCGTCGTTCCACTAGTGATTT-3' and inner primer: 5'-CGCGGATCCTCCACTAGTGATTTCACTATAGG-3') and miR-UL112-1 specific primer (5'-RGCCTGGRTCTCRCCGTCRCT-3'). The preparation of the reaction was conducted on ice. Reaction mixture was prepared as described by 3'-Full RACE Core Set. The first round amplification of hybrid-PCR was hot-started at 85°C, followed by 15-cycle amplification at an annealing temperature of 37°C. Extension was for 1.5 minutes. 1.5 μl of product from the first round amplification was used as templates in the second round PCR. The annealing temperature was increased to 55°C and the number of cycles to 25.

All PCR products were harvested by QIAEX ^® ^|| Gel Extraction Kit (Qiagen) and cloned into pMD-19T vectors (TaKaRa). Then plasmids were transformed into *E.coli *to produce a pool which should contain partial sequences of putative mRNAs that miR-UL112-1 would bind to. Clones were selected randomly. Insertions were identified by PCR using M13 primers, and checked by electrophoresis on 3% agarose gel to confirm the size of inserted fragments in the pool. Fifty-four clones, most of which were observed in different size, were picked and corresponding plasmids were sequenced on an ABI 3730 automated sequencer.

### Sequences blast and analysis

mRNA specific sequences located between the corresponding sequence of miR-UL112-1 hybrid-primer and polyA were intercepted and used to blast on line for identifying their host genes as putative target genes (http://www.ncbi.nlm.nih.gov/blast). Nucleotides in target sequences corresponding to miR-UL112-1 binding site were aligned with sequence of hybrid-primer respectively, in order to evaluate the complementary degree of miR-UL112-1 (especially of its seed region) to its target mRNAs.

### Plasmid construction

Six different target candidates were randomly chosen for validation by luciferase reporter assays. The 3'UTR of HCMV IE72 was used as positive control and the 3'UTR of HCMV IE86 was used as a true negative control. miR-UL112-1 putative binding sites within 500 bases of flanking sequences were amplified from mRNA-derived cDNA described above, and were then cloned into *SpeI *and *HindIII *sites of the luciferase reporter construct pMIR (Ambion) multiple cloning regions respectively. A 199-nucleotide-long sequence predicted to express miR-UL112-1 was cloned directly from genome of Han strain into miRNA expression vector pSilencer 4.1 (Ambion) at the *BamH I-Hind III *sites. Primer sequences used in plasmid construction were listed in Table 2. Expression of mature miR-UL112-1 was measured by TaqMan^® ^microRNA assays on 7300 Fast Real-Time PCR System (Applied Biosystems) (data not shown).

**Table 2:** Primer sequences used in plasmid construction

Genes inserted	Sequences
MiR-UL112-1	F: 5'-CGC**GGATCC**TCAGGTACTCGCAGGTGTGC
	R: 5'-CCC**AAGCTT**GTTGCCTGGACGCCTGGGCGCGA
HCMV IE72	F:5'-GG**ACTAGT**ACTATTGTATATATATATCAGT
	R:5'-CCC**AAGCTT**CGGTTTCACAGGCGTGACACGTT
Homo sapiens zinc finger protein 36, C3H type-like 1 (ZFP36L1)	F:5'-GG**ACTAGT**AGGCCTTTCACAACTAGGACTGA
	R:5'-CCC**AAGCTT**AAACTGCAAATAGTCGTTACAAA
Homo sapiens transportin 1	F:5'-GG**ACTAGT**TCTAATACACTTAAGCTGCAGT
	R:5'-CCC**AAGCTT**GCTTCTTCACATCCACTGCGGAGT
Homo sapiens ribosomal protein L7a	F:5'-GG**ACTAGT**GAAGACAAAGGCGCTTTGGCTA
	R:5'-CCC**AAGCTT**ATGTACAGAAAACTCAACAGT
Homo sapiens interleukin 32	F:5'-GG**ACTAGT**AGATACTGACACCACCTTTGCCCT
	R:5'-CCC**AAGCTT**CATGGTATCTCCCCTGCCAG
Homo sapiens mRNA for putative NFkB activating protein	F:5'-GG**ACTAGT**TGAACACAGAAAGTCTAAGAGGA
	R:5'-CCC**AAGCTT**GCTAATTAAACTTTGATTTTATTATG
HCMV UL17/18	F:5'-GG**ACTAGT**TACCAGCGGTTACGCACCGAG
	R:5'-CCC**AAGCTT**AACAGTTCCTCGGACATGATCA
HCMV IE86	F:5'-GG**ACTAGT**AGTCCACGGACCGCTCGGTCT
	R:5'-CCC**AAGCTT**TGCGCTCACCCGGCGTTCTC

Note: sequences recognized by restriction endonuclases are in bold.

### Luciferase reporter assays

Assays were conducted in a 24-well format. 200 ng pMIR construct carrying the putative target sequence was co-transfected into HEK293 cells along with 400 ng miR-UL112-1 expression plasmid and 200 ng control renilla plasmid pRL-TK (Promega) using Lipofectamine 2000 (Invitrogen) according to the manufacturer's recommendations. Plasmid (Ambion) that expressed a random small RNA was transfected as controls. Cells were collected 48 hours post transfection and luciferase activity levels were measured using the Dual luciferase reporter assay system (Promega) according to the manufacture's guidelines. All measurements were done in triplicates and signals were normalized for transfection efficiency to the internal Renilla control.

### Polymerase chain reactions

mRNA-derived cDNA above was amplified in another two reaction systems as described in the section for hybrid-PCR and sequencing, except that the initial annealing temperature was increased to 42°C and 55°C respectively. An additional PCR step was carried out with specific primers of target sequence to identify the seven validated target sequences (including IE72) among the hybrid-PCR products. Negative controls were created by adding no gene specific primers into PCR systems. Products were visualized by electrophoresis on 1.5% agarose gel.

## Competing interests

The authors declare that they have no competing interests.

## Authors' contributions

YJH carried out primer design, hybrid-PCR, PCR and sequence analysis. QR as the corresponding author designed the idea of the method and participated in revising the manuscript. YPM carried out virus preparation and cell culture, and YQ carried out RNA isolation and mRNA purification. RH and YHJ carried out plasmid construction. ZRS carried out luciferase reporter assays. All authors have read and approved the final manuscript.
